# Evolution of population dynamics following invasion by a non‐native predator

**DOI:** 10.1002/ece3.9348

**Published:** 2022-09-20

**Authors:** Sigurd Einum, Emil R. Ullern, Matthew Walsh, Tim Burton

**Affiliations:** ^1^ Centre for Biodiversity Dynamics, Department of Biology Norwegian University of Science and Technology Trondheim Norway; ^2^ Department of Biology University of Texas at Arlington Arlington Texas USA; ^3^ Norwegian Institute for Nature Research Trondheim Norway

**Keywords:** bottom‐up, density‐dependent selection, invasion ecology, predator–prey interactions, top‐down, zooplankton

## Abstract

Invasive predatory species are frequently observed to cause evolutionary responses in prey phenotypes, which in turn may lead to evolutionary shifts in the population dynamics of prey. Research has provided a link between rates of predation and the evolution of prey population growth in the lab, but studies from natural populations are rare. Here, we tested for evolutionary changes in population dynamics parameters of zooplankton *Daphnia pulicaria* following invasion by the predator *Bythotrephes longimanus* into Lake Kegonsa, Wisconsin, US. We used a resurrection ecological approach, whereby clones from pre‐ and post‐invasive periods were hatched from eggs obtained in sediment cores and were used in a 3‐month growth experiment. Based on these data, we estimated intrinsic population growth rates (*r*), the shape of density dependence (*θ*) and carrying capacities (*K*) using theta‐logistic models. We found that post‐invasion *Daphnia* maintained a higher *r* and *K* under these controlled, predation‐free laboratory conditions. Evidence for changes in *θ* was weaker. Whereas previous experimental evolution studies of predator–prey interactions have demonstrated that genotypes that have evolved under predation have inferior competitive ability when the predator is absent, this was not the case for the *Daphnia*. Given that our study was conducted in a laboratory environment and the possibility for genotype‐by‐environment interactions, extrapolating these apparent counterintuitive results to the wild should be done with caution. However, barring such complications, we discuss how selection for reduced predator exposure, either temporally or spatially, may have led to the observed changes. This scenario suggests that complexities in ecological interactions represents a challenge when predicting the evolutionary responses of population dynamics to changes in predation pressure in natural systems.

## Graphical abstract

In this study, we utilize an invasive event by the predatory spine water flea in combination with a resurrection ecology approach to investigate evolutionary changes in population dynamics of their main prey species (*Daphnia pulicaria*). Our results highlight that predators shape their communities not only through direct numerical effects on their prey, but also through evolutionary responses in prey population dynamics.

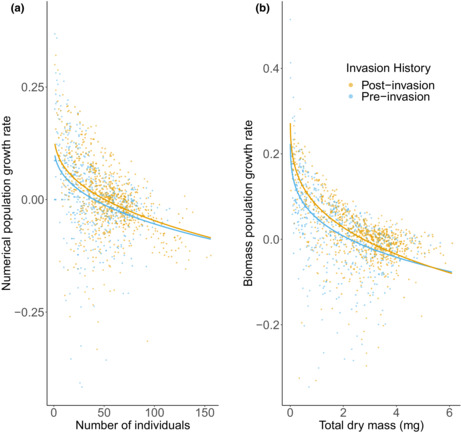

## 1 INTRODUCTION

A direct consequence of globalization is the facilitated movement of species to novel environments (Elton, 2020). Predatory species successfully invading an ecosystem are of particular concern, as they can have devastating effects on native prey species, often causing rapid changes in both population abundance and phenotypic traits (Reznick & Ghalambor, 2001; Strauss et al., 2006; Thompson, 1998). One obviously relevant aspect in this context is selective predation and resulting evolutionary responses in targeted traits (Åbjörnsson et al., 2004; Melotto et al., 2020). A less obvious aspect is that evolutionary responses in prey may also arise as an indirect byproduct of predator‐induced mortality. Such indirect selection may stem from large‐scale shifts in community structure and ecosystem function. For example, a shift in the direction of trophic control in the environment inhabited by the affected prey species may occur, from a state of ‘bottom‐up control’ (i.e. resource limitation) to ‘top‐down control’ (i.e. predator control), or vice versa. The resulting evolutionary responses in traits such as age at maturation, size and number of offspring, and competitive abilities may then be predicted based on density‐dependent selection theory (Einum et al., 2008; Mueller et al., 1991; Sæther et al., 2016). Indeed, evidence suggests that prey evolutionary responses resulting from such indirect selection can be of similar importance to those resulting from the direct selective mortality (Schmitz et al., 1997; Walsh & Reznick, 2010).

Given that predators may change prey phenotypes it follows logically that this could translate into evolution of their population dynamics. Such changes might include shifts in the intrinsic rate of increase and/or the strength of density dependence. Evolutionary responses in prey population dynamics have been demonstrated by rearing prey under different levels of predation under laboratory conditions (Shertzer et al., 2002; Turcotte et al., 2011; Yoshida et al., 2003). However, complimentary studies of evolved responses in natural populations are scarce (Walsh et al., 2012). Unfortunately, evolutionary response to predation can rarely be addressed by observing such phenomena in the wild, as ecological and evolutionary effects of the predator on population dynamics occur simultaneously and are confounded. For example, this has been considered to be a major constraint in studies of evolutionary responses of fish populations to harvesting (Heino et al., 2015). Ellner et al. (2011) provided an approach to disentangle the contribution from ecological and evolutionary effects in shaping changes in phenotypes of natural populations, but only for populations where pedigree information is available. An alternative approach is to compare the population dynamics of genotypes that have evolved under contrasting predation regimes in a common controlled environment. Bassar et al. (2013) employed such an approach to model population growth rate in guppies (*Poecilia reticulata*) based on demographic traits, and to compare modeled population growth across densities for populations originating from locations with different predation pressures. Although this approach has limitations in terms of extrapolating results to the wild (e.g. due to genotype‐by‐environment interactions), it can at the least demonstrate that a change in predation regimes of wild populations can translate into evolutionary effects on the intrinsic characteristics of the prey population dynamics, and thus corroborate previous studies (see above) that have demonstrated such evolutionary effects in artificial selection experiments. However, possibilities to study evolutionary change within single populations are limited to situations where (i) there is a documented change in predation regimes over time and (ii) genotypes from different time periods have been conserved and are available for experiments.

Here, we leverage the invasion of the North European spiny water flea (*Bythotrephes longimanus*), a predator of herbivorous zooplankton, into Lake Kegonsa, Wisconsin, US (Walsh et al., 2016). Since *Bythotrephes* was first detected at high abundance in this lake in 2009, the biomass of one of its prey species, the cladoceran *Daphnia pulicaria*, has been reduced by up to 60% (Walsh et al., 2016). In a previous study of *D. pulicaria* from this lake, Landy et al. (2020) compared resurrected clones (i.e. genotypes) originating from prior to the invasion of *Bythotrephes* with those of contemporary (i.e., post‐invasion) clones, and provided evidence that the invasion has led to evolutionary change in a suite of life history and behavioral traits. Specifically, they demonstrated that invasion by *Bythotrephes* was associated with evolved reductions in size at maturity and fecundity. In the current study, we follow‐up these findings and conduct population growth experiments in a common controlled environment to determine if the proliferation of *Bythotrephes* has led to evolutionary shifts in *D. pulicaria* population dynamics characteristics.

## 2 MATERIALS AND METHODS

### 2.1 Study animals and husbandry

Sediment cores containing ephippia of *Daphnia pulicaria* and live individuals were collected from Lake Kegonsa, Wisconsin, US (42.96°, −89.31°) in February 2018 and June 2019, respectively. For this study, eight pre‐invasion and eleven post‐invasion clones were used, most of which (17 out of 19) were a subset of those used by Landy et al. (2020) (Table A1). Sediment cores were ^210^Pb dated at the National Lacustrine Core Facility at the University of Minnesota, and *D. pulicaria* ephippia from pre‐ and post‐*Bythotrephes* invasion obtained from these cores were transported to the University of Texas at Arlington for hatching. Dormant eggs in *D. pulicaria* are a result of sexual reproduction, and only a single clone from each ephippium was used in the experiment. For live‐collected clones, these were obtained from multiple plankton tows that were taken from different locations within the lake early in the season (June) to minimize the risk of obtaining duplicate copies of the same clone. Hatched individuals from ephippia (representing all pre‐invasion and three post‐invasion clones, hatched during March 2019) and live‐collected individuals (representing post‐invasion individuals) were first kept at a 14 L:10D photoperiod at 16°C in 90 ml COMBO medium (Kilham et al., 1998) and fed non‐limiting supply of green algae (*Scenedesmus obliquus*, ~1.0 mg C L^−1^ day^−1^). In December 2019, live individuals of each clone were transported to the Norwegian University of Science and Technology and were subsequently kept at 17°C (photoperiod 16 L:8D) in ADaM medium (Klüttgen et al., 1994, SeO_2_ concentration reduced by 50%, sea‐salt increased to 1.23 g/L) and fed non‐limiting supply of Shellfish Diet 1800 (Reed Mariculture Inc.) until the onset of the experiment. The same medium, food, temperature and light regime was used throughout the rest of the study.

For each clone, 5–10 adult individuals were randomly chosen from stock cultures and placed in separate 1 L glass beakers (1 clone per beaker) where they were fed Shellfish Diet 1800 (Reed Mariculture Inc.) three times per week at a concentration of 4 ×x 10^5^ cells/ml. When several egg‐bearing individuals were identified in each beaker, these were isolated by removing all others. Beakers with egg‐bearing individuals were checked for neonates every 24 h and each neonate found within this period was individually transferred to a plastic container containing 100 ml of ADaM. Newborn individuals that died within 6 days were replaced using the same method. In total, 10 newborns were selected from each clone over a span of 7 days, yielding 190 populations that originally consisted of a single individual (10 individuals per clone × 19 clones, 8 pre‐invasion, 11 post‐invasion). When an individual died after the 7th day, it was reported as dead and not replaced (*n* = 11). In total, 21 pre‐invasion populations (23.3%) and 9 post‐invasion (9.0%) populations went extinct during the experiment, and 66.7% of these extinctions occurred within the first 10 days of the experiment. The plastic containers were stored in 2 Memmert Peltier cooled incubator IPP 260plus (Memmert, Germany) climate cabinets at 20.0°C (photoperiod 16 L:8D). Shellfish Diet 1800 (4 × 10^5^ cells/ml) was added every second day and medium was changed every 8 days. Container placement in the climate cabinets was changed haphazardly every 2 days, after feeding. All populations time series were run in parallel during March–May 2020.

### 2.2 Measuring population growth

To obtain data on population growth, video recordings were made of each population every 8 days (with one exception due to covid‐19 regulations) for a period of 3 months, starting 11 days after the start of the population growth experiment, creating a total of 10 censuses. From these videos, we estimated the number of individuals and the total dry biomass for each population in each census using the R package *trackdem* (Bruijning et al., 2018). Daily population growth rates, *G*, were calculated as *G* = log_e_(*N*_*t* + 1_/*N*_*t*_)/*d*, where *N*_*t*_ and *N*_*t* + 1_ is the population abundance (measured either in number of individuals or total biomass) at two consecutive censuses, and *d* is the duration of time in days between the censuses. For further details on these procedures, see Appendix 1.

### 2.3 Choice of population dynamics model and calculation of r

Inspection of population growth rate data (both for numerical and biomass growth) revealed strong non‐linearity in the density dependence. Thus, the population dynamics is best described by the theta‐logistic model *G* = *r*(1−[*N*_*t*_/*K*]^*θ*^) where *r* is the intrinsic population growth rate, *K* is the carrying capacity, and *θ* determines the shape of the density dependence. One concern when fitting this model to data is that different combinations of *r* and *θ* can produce model fits of similar likelihood, potentially resulting in ecologically unrealistic estimates of both parameters (Clark et al., 2010). We therefore took advantage of the experimental design, whereby each population was started with a single neonate, which allowed us to obtain direct observations of population growth under low density. We based this calculation of *r* for each population on the observed population abundance at the start of the experiment (*N*_0_) and the second census (*N*_2_, i.e. *r* = log_e_[*N*_2_/*N*_0_]/*d*). This duration (i.e. *d* = 18 days) is similar to the standard time span of 21 days used for quantification of reproductive rates in *Daphnia* (OECD, 2012). This was done both for numerical and biomass growth.

### 2.4 Statistical analyses

All statistical analyses were conducted in R v.4.1.0 (R Core Team, 2021). We tested for an effect of invasion history on the observed values of *r* (calculated early in the sequence of population growth and thus under low density, see above) by fitting a linear mixed model to these data using the function *lme* in the package *nlme* (Pinheiro et al., 2021), including a random effect of clone ID, and comparing this against a simpler model containing only the random effect using the Akaike information criterion corrected for small sample sizes (*AICcmodavg*, Mazerolle, 2020). This was done both for numerical and biomass growth rate.

Next, to test for an effect of invasion history on *K* and *θ*, we fitted non‐linear mixed effect models representing the theta‐logistic model to the population growth time series. This was done for the data following the first two censuses, i.e. after the period that had been used to calculate *r*. Again, this was done separately for numerical and biomass growth rates. In the full model, population growth rate over a given period (between two consecutive censuses) was modeled as a function of the observed value of *r* for that population and its population size at the start of that period while estimating values of *K* and *θ* that depended on invasion history. The model included random effects of clone ID and population nested within clone ID on *K*. The model was fitted using the function *nlme* in the package *nlme* (Pinheiro et al., 2021). This full model was compared with a simpler one where *K* and/or *θ* were common to all populations independent of invasion history, again using the Akaike information criterion corrected for small sample sizes. For all analyses, assumptions of normality and homogeneity of residuals were satisfied. All figures were made using the package *ggplot2* (Wickham, 2016).

## 3 RESULTS

The population dynamics of most clones consisted of an initial increase in abundance up to a peak value, followed by a subsequent decline (Figure A1). According to the model comparison, observed values of intrinsic population growth rate tended to depend on invasion history (Table 1). The evidence for such an effect in terms of ΔAIC was strongest for biomass (Table 1), but models containing an effect of invasion history suggested higher intrinsic population growth rate in post‐invasive clones than in pre‐invasive clones for both numerical and biomass growth (Table 2). The estimated increase in intrinsic population growth rate based on numerical and biomass data were 23% and 15%, respectively (Table 2).

**TABLE 1:** AICc comparisons of candidate models explaining variation in observed intrinsic population growth rate (*r*) carrying capacity (*K*) and the shape of density dependence (*θ*) of experimental populations of *Daphnia pulicaria* originating from Lake Kegonsa. For *r*, separate linear mixed effects models are fitted to numerical and biomass population growth rate as dependent variables. Full models include effects of invasion history (whether the population originates from a period before or after invasion by the predatory zooplankton species *Bythotrephes longimanus*), with clone ID as a random effect. For *K* and *θ*, separate theta‐logistic models are fitted to numerical population growth rate and biomass population growth rate as dependent variables. Full models include effects of invasion history on *K* and *θ*, with clone ID and population (nested within clone ID) included as random effects.

	*k*	AIC_C_	ΔAIC_C_	*w*_*i*_
Numerical, population growth rate				
*r* ~ invasion history	4	−650.61	0.00	0.61
*r* ~ 1	3	−649.68	0.93	0.39
Biomass, population growth rate				
*r* ~ invasion history	4	−598.93	0.00	0.73
*r* ~ 1	3	−596.94	1.98	0.27
Numerical, carrying capacity and theta				
*K* ~ invasion history, *θ* ~ 1	6	−3010.67	0.00	0.56
*K* and *θ* ~ invasion history	7	−3010.00	0.67	0.40
*K* and *θ* ~ 1	5	−3004.93	5.73	0.03
*K* ~ 1, *θ* ~ invasion history	6	−3003.48	7.19	0.02
Biomass, carrying capacity and theta				
*K* and *θ* ~ invasion history	7	−3314.99	0.00	0.39
*K* ~ invasion history, *θ* ~ 1	6	−3314.12	0.86	0.25
*K* ~ 1, *θ* ~ invasion history	6	−3314.04	0.95	0.24
*K* and *θ* ~ 1	5	−3312.70	2.29	0.12

**TABLE 2:** Parameter estimates (obtained using REML) of the best fitting models describing the variation in observed intrinsic population growth rate (*r*) of *Daphnia pulicaria* originating from Lake Kegonsa (Table 1). Post‐and pre‐invasion populations consist of clones originating from after and before *Bythotrephes longimanus* invasion, respectively.

Numerical	Estimate	*SE*	*p*
Fixed effects			
*r* post‐invasion (intercept)	0.16	0.01	
*r* pre‐invasion (difference)	−0.03	0.02	.0977
Random effects (*SD*)			
Clone ID	0.04		
Residual	0.03		
**Biomass**			
Fixed effects			
*r* post‐invasion (intercept)	0.31	0.01	
*r* pre‐invasion (difference)	−0.04	0.02	.0560
Random effects (*SD*)			
Clone ID	0.04		
Residual	0.03		

**FIGURE 1:**
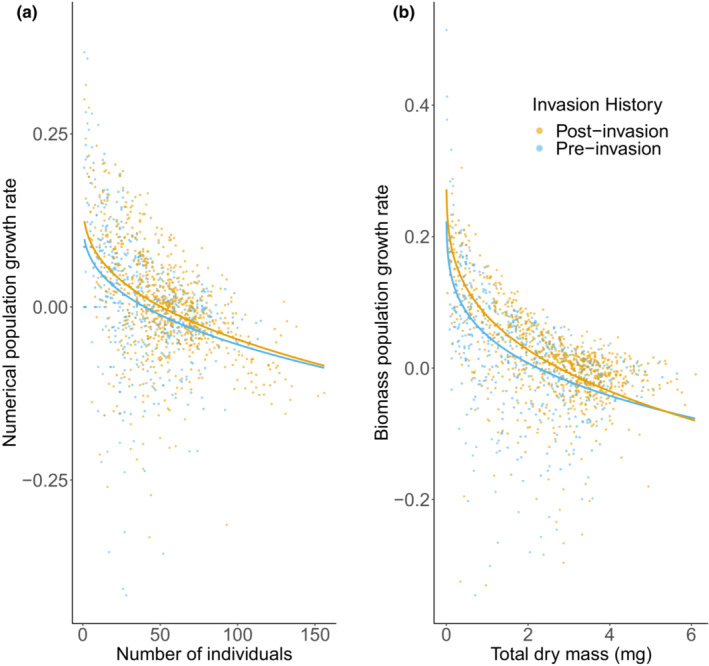
Growth rate in terms of (a) number of individuals and (b) total dry mass for pre‐ and post‐invasion populations of *Daphnia pulicaria* originating from Lake Kegonsa. Pre‐ and post‐invasion populations consist of clones sourced from before and after *Bythotrephes longimanus* invasion in 2009, respectively. Regression lines give predictions from theta‐logistic models with parameter estimates from Table 2 (for *r*) and Table 3 (for *K* and *θ*).

The difference in population dynamics between pre‐ and post‐invasion clones was also reflected in the comparisons of theta‐logistic models of population growth rates. Particularly for numerical data, the model comparison shows strong evidence for an effect of invasion history on *K* (Table 1). Evidence for effects of invasion history on *θ* was weaker for these data, and the best model did not contain such a term (Table 1). For biomass data, the best model contained effects of invasion history on both *K* and *θ* (Table 1). Models containing such effects on only one of these two parameters received slightly less support, whereas a model containing no effect of invasion history received considerably less support (Table 1). For both these analyses, the best models predicted a higher carrying capacity for post‐invasion clones compared with for the pre‐invasion clones (Table 3, Figure 1). The estimated increase in carrying capacity based on numerical and biomass data were 27% and 23%, respectively (Table 3).

**TABLE 3:** Parameter estimates (obtained using REML) for *K*, *θ* and random effects of *K* of the best fitting theta‐logistic models describing the population dynamics of *Daphnia pulicaria* originating from Lake Kegonsa (Table 1). Post‐and pre‐invasion populations consist of clones originating from after and before *Bythotrephes longimanus* invasion, respectively.

Numerical	Estimate	*SE*	*p*
Fixed effects			
*K* post‐invasion (intercept)	50.88	2.11	
*K* pre‐invasion (difference)	−10.89	3.08	.0004
*θ*	0.38	0.03	
Random effects (*SD*)			
Clone ID	0.0033		
Population:Clone ID	0.0011		
Residual	0.0763		
**Biomass**			
Fixed effects			
*K* post‐invasion (intercept)	2.76	0.17	
*K* pre‐invasion (difference)	−0.52	0.28	.0619
*θ* post‐invasion (intercept)	0.29	0.02	
*θ* pre‐invasion (difference)	−0.04	0.02	.0706
Random effects (*SD*)			
Clone ID	0.5069		
Population:Clone ID	0.0001		
Residual	0.0672		

## 4 DISCUSSION

We leveraged a well‐documented invasive event by a predatory species (*Bythotrephes longimanus*) in combination with a resurrection ecology approach to investigate evolutionary changes in population dynamics of their main prey species (*Daphnia pulicaria*). This allowed us test for an association between a change in predation levels and evolution of prey population dynamics in a natural population. Estimation of population dynamics parameters showed that post‐invasion genotypes had an increased intrinsic population growth rate *r* (strong evidence for biomass data) as well as carrying capacity *K* (strong evidence for numerical data). For the current study, we had prior information on evolutionary change in individual traits in response to the predator invasion. Specifically, for traits that can be directly used to predict effects on population dynamics, Landy et al. (2020) found that post‐invasion clones had a reduced size at maturity and reduced fecundity compared with pre‐invasion clones, but found no significant difference in age at maturity. Based on this, one might predict a reduced intrinsic rate of increase in post‐invasive clones. Yet, we observed the opposite in our study. One reason for this could be that unmeasured traits that may be genetically correlated with those that were measured may also have influenced population dynamics. For example, if offspring quality and survival is negatively correlated with fecundity (Mappes & Koskela, 2004), these two traits may counteract each other in terms of effects on population growth.

Although there is abundant evidence that changes in predator communities can lead to pronounced evolutionary change in the behavior, morphology and life‐history traits of their prey (including work on resurrected *Daphnia*, Stoks et al., 2016), we are not aware of previous studies that have quantified the evolutionary effect of predation level on the intrinsic population dynamics of wild prey populations. However, a series of chemostat experiments have addressed the role of evolution in shaping the population dynamical response of planktonic algae (*Chlorella vulgaris*) to rotifer (*Brachionus calyciflorus*) predation. These studies have demonstrated that the patterns of predator–prey cycles are influenced by prey evolution (Shertzer et al., 2002; Yoshida et al., 2003). This system has also been used to test for an evolutionary trade‐off between algal population growth and predator defense (Kasada et al., 2014; Meyer et al., 2006; Yoshida et al., 2004). Yoshida et al. (2004) allowed algae to evolve in the presence and absence of the rotifer, after which their growth rates were measured at different nutrient‐levels in the absence of predation. The results showed that algae that had evolved under predation exposure had a lower population growth rate than those having evolved in absence of predation, but only at the most limiting nutrient‐level. When nutrients were more abundant no such difference was observed. Thus, in this system it appeared that adaptation to predation primarily caused a reduction in *K*, with no effect on *r*.

In the current study, we found no indication of an interaction between predation history and food availability in determining the rate of population growth as observed in the rotifer/algae experiments. If such an interaction had been present, we would expect the relative difference in population growth rate between the two types to differ at low and high population density, which in turn should translate into different effects of type on *r* and *K*. Instead, models that contained an effect of invasion history consistently suggested elevated values of both *r* and *K* in post‐invasion clones compared with pre‐invasion clones, independent of measurement type (numerical or biomass population dynamics), meaning that post‐invasion clones showed weaker effects of numerical density (and per capita food availability) on per capita growth rate when compared with pre‐invasion clones, with slightly higher overall growth rates. This may indicate that evolutionary responses to invasive predators in natural systems can be complex. One caveat of the result in this study is that they are obtained in a laboratory environment, with different biotic (e.g. competition, parasites, and food quality) and abiotic (e.g. temperature, water chemistry) conditions than what this population experience in the wild. Specifically, in the presence of genotype‐by‐environment interactions, a given phenotypic difference between two genotypes observed in one environment may disappear or even be reversed in a different environment. This is a general concern with such common environment experiments that aim to describe genetically based differences in phenotype. A second caveat is that whereas all pre‐invasion clones were derived from ephippia, and thus represent a sub‐set of clones that would hatch at the onset of season in the spring, most of the post‐invasion clones were collected as live individuals, at which time some changes in the population's genetic composition may have occurred due to selective predation. We attempted to minimize such an effect by conducting the sampling early in the season (early June), at which point the population should have experienced limited predation by *Bythotrephes* which have a much slower population growth and peak in abundance in October (Walsh et al., 2016). However, we cannot exclude the possibility that the clonal composition at the time of sampling of live individuals had become different from that in the resting egg stage due to clonal differences in population growth during the early season. If so, rapidly growing clones with a high *r* may then have been overrepresented in our sampled live clones. Although limited sample size precludes meaningful statistical comparisons of post‐invasion clones originating from ephippia (*n* = 3) vs. live‐collected ones (*n* = 8) to test for an effect of collection methodology, inspection of clone‐specific population dynamics does not indicate a consistent difference in population dynamics between these two groups (Figure A1).

We propose two potential explanations for the apparent counterintuitive result that post‐invasive clones have both a higher *r* and *K*. First, as for most zooplankton, both species show extensive seasonal dynamics in abundance, but they are not synchronous. Data from nearby Lake Mendota, another lake having experienced a recent *Bythotrephes* invasion, show that whereas *D. pulicaria* population abundance peaks during early summer (May/June), *Bythotrephes* populations have a low population abundance early in the season and peak several months later (see above). Thus, the invasion and associated heavy predation toward the last part of the growth season may have strengthened selection for rapid growth, favoring clones that reach high abundance prior to the onset of high predation rates. We do not have data on the timing of resting egg production in Lake Kegonsa, which would indicate the importance of reaching high frequency early in the season. However, sampling in late September 2018 showed that *D. pulicaria* was largely absent by that time (M. Walsh, unpublished data), suggesting that the largest contribution to the resting egg bank may occur during summer.

A second potential explanation relates to the propensity for *D. pulicaria* to migrate vertically in the presence of a predator, whereby they move to deeper parts of the lake during the day to avoid predation. Indeed, Landy et al. (2020) found evolution toward reduced positive phototaxis in post‐invasive clones from Lake Kegonsa, suggesting an increased propensity to undertake such migrations. *Daphnia* feed on phytoplankton, which tend to congregate at the lake surface. Thus, life in deeper water also means living in a more resource limited environment (Cousyn et al., 2001; Pangle & Peacor, 2006). Previous studies have shown that organisms living at different resource levels may evolve adaptations to this, such that when reared in a common environment, those from more food‐restricted environments actually grow faster (Arendt & Wilson, 1999). Thus, our observation that post‐invasive clones outperform pre‐invasive clones may be an example of such countergradient variation (Conover & Schultz, 1995), where they have evolved physiological adaptations that increase population growth under a given level of food abundance. In a recent study of *D. pulicaria* from Lake Mendota, Rani et al. (2022) found that post‐invasive clones had a reduced metabolic rate compared with pre‐invasion clones, which may represent one such physiological adaptation to a cooler resource deficient environment. Furthermore, Einum et al. (2019) found that variation in somatic growth rate among clones of *D. magna* was best explained by clone‐specific food consumption expressed relative to their rate of energy use. Thus, if post‐invasion clones have a reduced rate of metabolism but do not moderate food consumption when reared in a common environment (as in the current study), this could be expected to result in increased somatic growth rate and may thus explain the higher population growth rate.

To conclude, the current study demonstrates an evolutionary shift in the population dynamics of *D. pulicaria* in parallel with an increase in predation brought about by invasion of the predatory zooplankton *Bythotrephes*. To our knowledge, this is the first empirical study that directly demonstrates this by comparing genotypes of a single natural population that has experienced a temporal change in exposure to predation. Although the potential for genotype‐by‐environment interactions prevents strong conclusions regarding fitness of pre‐ and post‐invasion clones in the wild, we suggest that the observed shift in population dynamics may be related to selection for reduced predator exposure, either temporally or spatially. If so, this suggests that complexities in ecological interactions represents a challenge when predicting the evolutionary responses of population dynamics to changes in predation pressure in natural systems.

## AUTHOR CONTRIBUTIONS

**Sigurd Einum:** Conceptualization (equal); formal analysis (equal); funding acquisition (equal); investigation (supporting); methodology (equal); project administration (lead); resources (equal); supervision (equal); writing – original draft (equal); writing – review and editing (lead). **Emil R. Ullern:** Data curation (lead); formal analysis (equal); investigation (lead); methodology (supporting); writing – original draft (equal); writing – review and editing (supporting). **Matthew Walsh:** Conceptualization (equal); resources (supporting); writing – review and editing (supporting). **Tim Burton:** Conceptualization (equal); formal analysis (supporting); funding acquisition (equal); methodology (equal); project administration (supporting); resources (equal); software (lead); supervision (equal); writing – review and editing (supporting).

## CONFLICT OF INTEREST

The authors declare that they have no conflict of interest.

### OPEN RESEARCH BADGE

All data used in this study are provided as private‐for‐peer review at https://figshare.com/s/7e33eed4ecf3c36e56fe. These data will be made publicly and permanently available at Figshare upon acceptance for publication.

## Supporting information


**Appendix 1** Supporting information

## Data Availability

All data used in this study are provided as private‐for‐peer review at https://figshare.com/s/7e33eed4ecf3c36e56fe. These data will be made publicly and permanently available upon acceptance for publication.
