# Supplementary material for: Evolution of population dynamics following invasion by a non‐native predator
Source: Ecol Evol. 2022 Sep 20;12(9):e9348. doi: 10.1002/ece3.9348 (PMC9487876; doi:10.1002/ece3.9348)
Supplement: Supplementary file 1 — Appendix 1 Supporting information [file ECE3-12-e9348-s001.docx]

# Appendix 1

# **Table A1**: Clone ID of individuals used in this study. Clones with “KC” in their ID were collected from the water column whereas the remaining clones were hatched from ephippia gathered from sediment cores. Test individuals are *Daphnia pulicaria* originating from Lake Kegonsa (Wisconsin, USA) reared in laboratory conditions at NTNU from March to June 2020. Post-invasion populations consist of clones stemming from after *Bythotrephes longimanus* invasion in 2009. Pre-invasion populations consist of individuals stemming from before 2009. * denotes clones that were used by Landy et al. (2020).

| Clone ID | Estimated year of origin | Invasion history |
| --- | --- | --- |
| K28_B* | 1990 | Pre-invasion |
| K26_A* | 1993 | Pre-invasion |
| K25_C* | 1994 | Pre-invasion |
| K25_B* | 1994 | Pre-invasion |
| K24_A* | 1996 | Pre-invasion |
| K22_A* | 1999 | Pre-invasion |
| K16_A* | 2006 | Pre-invasion |
| K15_A* | 2007 | Pre-invasion |
| K12.5_C* | 2010 | Post-invasion |
| K9.0_B* | 2013 | Post-invasion |
| K1.5_P | 2017 | Post-invasion |
| KC150* | 2019 | Post-invasion |
| KC201* | 2019 | Post-invasion |
| KC203 | 2019 | Post-invasion |
| KC45* | 2019 | Post-invasion |
| KC52* | 2019 | Post-invasion |
| KC59* | 2019 | Post-invasion |
| KC64* | 2019 | Post-invasion |
| KC80* | 2019 | Post-invasion |

**Filming and estimation of population sizes and growth rates using *trackdem***

To prepare each population for filming, the content of each container was poured into a second plastic container fitted with a fine mesh sieve to remove most of the medium. *Daphnia* were subsequently flushed (with ADaM) from the sieve with the help of a spraying bottle into a transparent square tray (9.0×9.0cm). Large pieces of algae were removed from the tray by pipetting. The empty plastic container was also sprayed with ADaM to remove attached algae and leftover *Daphnia*. This content was poured into a second tray and if *Daphnia* were found, these were moved with a pipette to the main tray.

The transparent tray was put on a LED light board (Huion A4 LED light pad, set to maximum intensity) in a dark room directly under the camera (Basler aCA1300-60gm, fitted with 5–50-mm, F1.4, CS mount lens) and filmed for 10-14 seconds. We waited for any movement of the medium to stop before starting each recording. The contents of the tray were then emptied back in the original plastic container. This procedure was repeated for each population. After finishing, each container was filled to 100mL with ADaM and 1.5 mL shellfish diet was added. The larger ration during this event than during regular feeding was done to compensate for the complete removal of all algae and bacteria during medium change. The procedure described above produced a total of 1657 videos that were subsequently analyzed to obtain data on population sizes. The first 39 frames from each video were extracted as images using a custom Matlab script. The images were then analyzed using the R package *trackdem* (Bruijning et al., 2018). *trackdem* estimates the number of live individuals (based on movement and contrast) and their size (based on number of pixels) in a sequence of images. Dry mass (mg) of each individual was calculated as -0.00635 + 0.00100 x pixel number, based on a regression developed for *D. magna* (Fossen et al., 2021). Population sizes from 18 sample videos selected from throughout the experimental period were counted manually and optimal *trackdem* settings were found by regressing these against estimated counts from *trackdem*. For the optimal settings (see below), the fitted regression was *trackdem* count = 0.08 + 0.96 x manual count (R^2^ = 0.93, n = 18).

Growth rate was not calculated between two censuses if the population went extinct. Outliers were identified by inspecting residuals from a linear regression between population count and growth rate. Manual inspection of videos with high residual values identified 16 instances where *trackdem* estimates of population size were unexpectedly large. These values were corrected by conducting manual counts from the respective videos. All of these cases were caused by a high abundance of moving algae, mistakenly counted as *Daphnia* by *trackdem*. Manual inspection of videos producing negative outliers were confirmed to be valid and caused by rapid population declines.

***trackdem* settings**

*partIden: pixelRange=c(5,1000)*

After looking at outputs from *trackdem* and comparing them to a manual analysis of the corresponding videos, actual daphnia recorded by the software were never below 5 pixels in size. *Trackdem* did, however, count floating debris (size <5) as daphnia and the pixel range was therefore set to ≥ 5 to avoid this. The upper limit to the range (≤1000) has no impact on the output.

*partiden: threshold=-0.12*

Contrast threshold for including particles in the count. -0.1 is the default threshold for dark particles against a light background (<https://cran.r-project.org/web/packages/trackdem/trackdem.pdf>) -0.12 was chosen after testing different thresholds for one randomly selected video.

*records: trackParticles(partIden,L=70,R=5, weight = (1,2,0), costconstant=TRUE,logsizes=TRUE)*

*L*: Maximum cost for linking a particle to another particle. When the cost is larger, particles will be not be linked (resulting in the beginning or end of a segment). Default set at 50 (<https://cran.r-project.org/web/packages/trackdem/trackdem.pdf>). L was set to 70 to avoid linking of daphnia who would swim over each other for short periods of time.
*R*: The maximum number of frames set to which particles after frame N can be linked. Default is set to 2 (<https://cran.r-project.org/web/packages/trackdem/trackdem.pdf>). 5 was chosen because of the twitching movement of daphnids causing them to remain still for periods of time.
*Weight*: Weights are ordered as follows; first number gives the weight for differences in x and y coordinates; second number gives the weight for particle size differences; third number gives the difference between the predicted location and the observed location. Default is set to (1,1,1). (1,2,0) was chosen because: (**1**,2,0) no reason was found to not keep it default, (1,**2**,0) daphnia change their 2D surface area when they swim which causes their representing particle to change size, (1,2,**0**) no prediction was best since daphnia change directions and accelerate rapidly.
*costconstant=TRUE*: Set to *TRUE* keeps maximum cost *L* constant for all 1:R frames. Adapted for situations where individuals do not move in and out of the frame but may overlap and lay still for several seconds. Meant to avoid the creation of new segments when an individual is behaving like previously mentioned.
*logsizes*=*TRUE*: ln of body size is used when calculating the cost of linking two particles for a more representable, proportional change in size.

*MeanSize/SdSize/Movement: as.vector(summary(records,incThres=0.4*nr.pictures)*

IncThresh determines the minimum number of frames a particle must be present to be counted. The value 0.4 was chosen after comparing linear regression models with different values.

*Movement threshold: movement ≥40*

Threshold set to only register individuals with a movement of ≥40 pixels. The threshold was set to exclude as much debris as possible (moving algae as false positives) while at the same time not exclude live animals. The value was selected after comparing linear regression models with different relevant values.


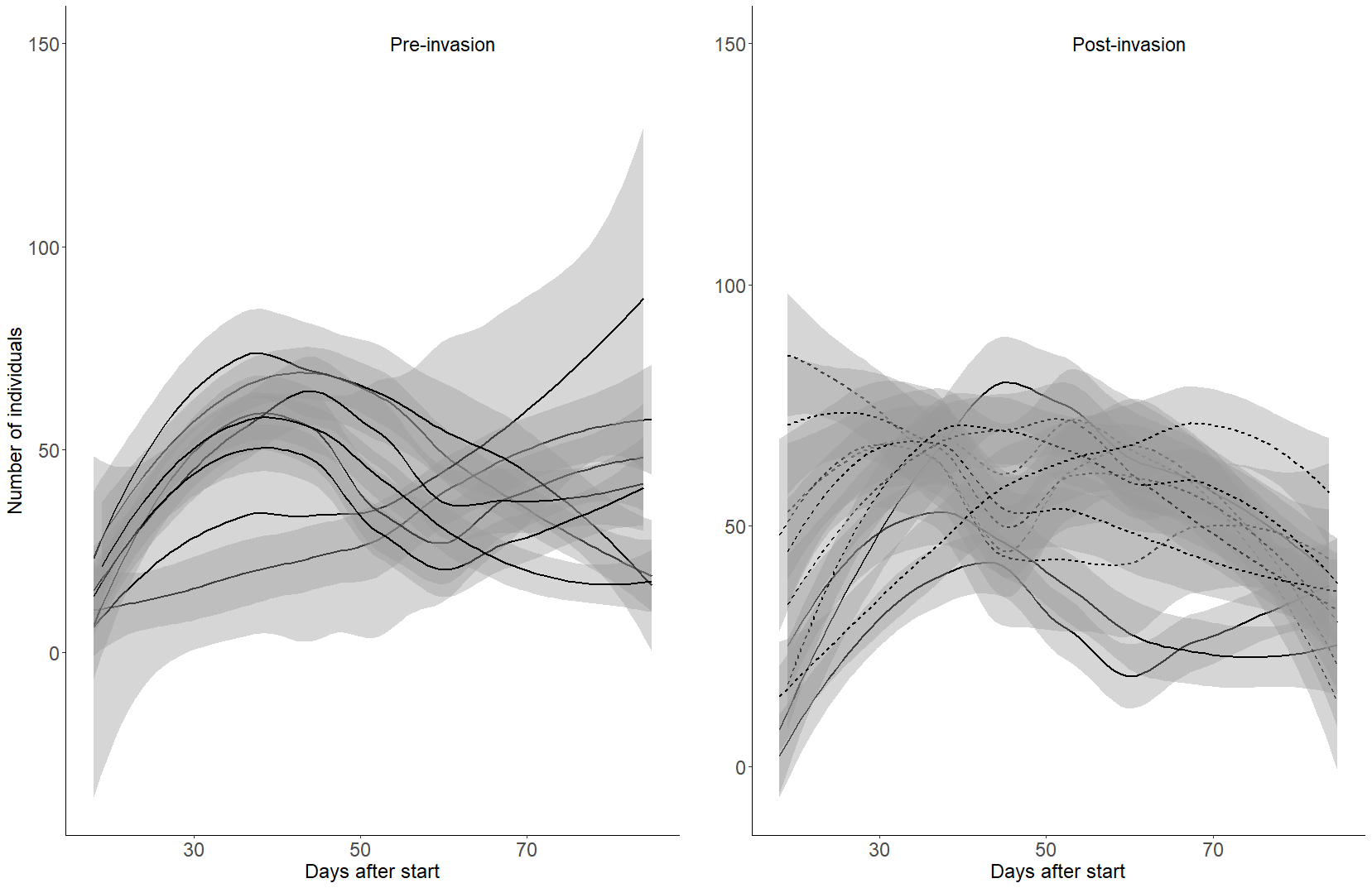


Fig. A1. Trends in population abundances throughout the experiment for different clones of *D. pulicaria* originating from prior to or after the invasion by *B. longimanus*. Each clone is represented by a separate line (drawn using the geom_smooth function), with shaded areas giving 95% confidence intervals. For post-invasion clones, solid lines represent clones originating from ephippia, whereas dashed lines represent live-collected clones.

**References**

Bruijning, M., Visser, M.D., Halllmann, C.A., Jongejans, E. (2018). trackdem: Automated particle tracking to obtain population counts and size distributions from videos in r. *Methods in Ecology and Evolution,* 9, 965-973.

Fossen, E.I.F., Raeymaekers, J.A.M., Einum, S. (2021). Do genetic differences in growth thermal reaction norms maintain genetic variation in timing of diapause induction? *Freshwater Biology*, <https://doi.org/10.1111/fwb.13825>.

Landy, J.A., Oschmann, A., Munch, S.B., Walsh, M.R. (2020). Ancestral genetic variation in phenotypic plasticity underlies rapid evolutionary changes in resurrected populations of waterfleas. *Proceedings of the National Academy of Sciences*, 202006581.
